# Cadmium Status Among Pediatric Cancer Patients in Egypt

**DOI:** 10.1097/MD.0000000000000740

**Published:** 2015-05-22

**Authors:** Laila M. Sherief, Elhamy R. Abdelkhalek, Amal F. Gharieb, Hanan S. Sherbiny, Doaa M. Usef, Mohamed A.A. Almalky, Naglaa M. Kamal, Mostafa A. Salama, Wafaa Gohar

**Affiliations:** From the Department of Pediatrics, Faculty of Medicine, Zagazig (LMS, ERA, HSS, DMU, MAAA, WG); Cairo (NMK); Benha Universities (MAS); Benha children Hospital, Benha (LMS, ERA, MAS); and Department of Biochemistry, Zagazig University, Zagazig, Egypt (AFG).

## Abstract

Cadmium (Cd) is a toxic, nonessential, and bio-accumulating heavy metal widely used in industry. Several studies have suggested a positive association between Cd exposure and risks of several cancers. However, data from general population, especially children are sparse.

In the current cross-sectional case–control study, we aimed to assess the association between Cd exposure, as expressed by Cd body status (blood, urine, scalp hair, and nails) and cancer among Egyptian children. Three hundred and fifty pediatric cancer cases aged 3 to 14-years old were enrolled in our study. Their body Cd levels were evaluated using Atomic Absorption Spectrophometer and were compared with Cd levels of 350 healthy children.

Significantly higher Cd levels (blood, urine, scalp hair, and nails) were documented in cancer cases when compared with control (*P* < 0.001). Such difference was still detected when comparing each malignant type separately, with controls. Tobacco smoke exposure, rural residence, and low socioeconomic status were reported more frequently among cases than comparisons.

Positive association between Cd exposure and pediatric malignancy may be present.

## INTRODUCTION

Cancer is relatively rare in children, approximately 1.2 cases/10,000 children ≤14 years are diagnosed in the United States each year.^1^ Environmental causes of cancer, as used by cancer researchers, mean any cause that is not genetic, environmental pollutants represent an important aspect of them.^2^ In 1993, The International Agency for Research on Cancer (IARC) has classified cadmium (Cd) as human carcinogen based on studies in occupational exposed workers.^3^

Cd is a natural element in the earth crust, all soils and rocks contain some Cd. It does not erode easily and has many uses including batteries, pigment, metal coating, and plastic, it is also a contaminant of some commercial fertilizers.^4^ The environment is polluted by Cd through emission from industrial activities, agricultural practices using contaminated phosphate fertilizers and sewage sludge, and by tobacco smoke.^5^ Cd emitted to the atmosphere from combustion process is usually associated with very small particulates, which are in the respirable range and subject to long-term transport.^6^ Cd dissolves to some extent in water and binds strongly to soil, it can easily be taken by some plants (leafy vegetables) and contaminates the food chain.^7^ Once Cd is emitted to the environment, it is transported continuously between the 3 main environment compartments air, water, and soil. Its compounds do not evaporate or disappear from the environment.^8^

Children are exposed to Cd mainly by food intake and by inhaling air, which is polluted by environmental tobacco smoke, house dust, and industrial emission.^9^ Excretion from the body is limited, the biological half-life is reported to be 10 to 30 years in the kidneys and 4.7 to 9.7 years in the liver. The WHO reported tolerable weekly intake of Cd as 0.007 mg/kg body weight for all human groups,^10^some authors believe that this metal can dangerously affect human health even at ultra-trace concentration.^11^

Several epidemiologic and laboratory studies have suggested a direct relation between Cd exposure and the risk of several cancers,^12–14^ while others have found no such association.^15–17^ Few population-based studies have addressed the association between cancer and environmental Cd exposure and have reported inconsistent results.^18,19^ Very few Cd-associated health effect studies in children exist. As Cd exposure and accumulation in the body start at young age,^20^ the aim of the current study is to assess the association between Cd exposure, as expressed by Cd levels in different body samples (blood, urine, scalp hair, and nails), and cancer among Egyptian children and also to explore the possible sources of its exposure.

## PATIENTS AND METHODS

### Patients

We carried this cross-sectional case–control study on 350 pediatric cancer patients aged 3 to 14-years old, from those attending the pediatric oncology clinics in 3 hospitals namely Zagazig University Hospital, Tanta Cancer Institute, and Benha children Pediatric Hospital-Egypt during the period from January 2011 to May 2014. Our target population were children of both sex with pathologically confirmed malignancy of any type (both hematologic and solid malignancies were included). From each contributing hospital, we identified a random population sample stratified by age and sex (they attended the hospitals for follow-up of simple medical or surgical disorders) with the aim of recruiting 350 children as controls. The study protocol was approved by the research and ethical committee of the contributing hospitals, and informed consent was taken from the parent or guardian of each enrolled child.

### Methods

All eligible children (cases and controls) were subjected to, history taking including, demographic characteristics, residency, and tobacco exposure. Thorough physical examination, routine investigations, and specific tests for evaluating Cd status in their bodies including serum Cd (S-Cd), spot urine Cd (U-Cd), and Cd levels in both scalp hairs and nails, were performed.

S-Cd and U-Cd level in spot urine sample (10 mL) were performed using Atomic Absorption Spectrophometer GBC model with wavelength 2280.8 nm, working range 0.2 to 1.8 with sensitivity 0.0009 (μg/L). Urine was taken at various times of the day from the participants, as studies of timed urine samples indicated that urinary Cd does not show significant diurnal variation.^21^ Cd was also evaluated at scalp hair and nail samples, all samples were whole oven dried, and were pulverized into fine texture, dissolved in nonionized water in digestion flask, and digested with mixture of 10 mL of nitric acid and 2 mL of concentrated perchloric acid. Cd estimation was performed with using Atomic Absorption Spectrophometer VGP BUCK scientific USA model 210 with an oxidizing air acetylene flame (A.O.A.C, 1990). Cd assays were carried out at Central Laboratory of Biochemistry, Zagazig University, Egypt. All results were expressed as μg/L.

Definitions: exposure to smoking, as smoking is a major cause of Cd exposure in the general population and Cd is a cumulative toxicant, participants were classified as tobacco smokers exposed if they had ever used and/or household exposed to person who used, smoking materials.^22^ Socioeconomic status was classified according to UK office of population censuses and survey low (unskilled occupation), medium (manual and nonmanual skilled occupation), and high (professional occupation).^23^ Living near industrial facility was considered if resident within 10 km distance from nonferrous manufacture factory, combustion of oil product, waste incineration, or battery manufacturing.^22^

## STATISTICAL ANALYSIS

Analysis of data was done using statistical program for Social Science version 15 (SPSS Inc., Chicago, IL). Quantitative (numerical) variables were described in the form of ranges, means, and standard deviations while categorical variables were described as number and percentages. For normally distributed variables, parametric test like student *t* test was used for comparing 2 groups. As Cd levels did not fulfill criteria for normal distribution, Mann–Whintey test was used for comparing 2 groups while Kruskall–Wallis test was used for comparing more than 2 groups. For all previous statistical tests done, *P* value of <0.05 indicated statistical significance.

## RESULTS

**FIGURE 1:**
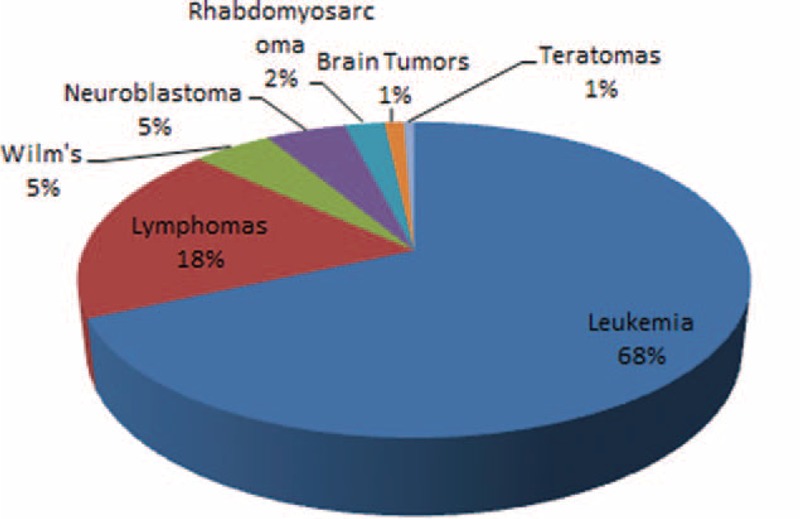
Prevalence of different types of malignancy.

A total of 350 children with confirmed malignancies were recruited in our study, leukemia was the leading diagnosis (239/68.3%) followed by lymphoma (65/18.6%). Wilm tumor and neuroblastoma were diagnosed in (16/4.6%) each, while rhabdomyosarcoma, brain tumors, and teratomas were diagnosed in (8/2.3%, 4/1.1%, and 2/0.6%) cases, respectively, as shown in Figure 1.

**FIGURE 2:**
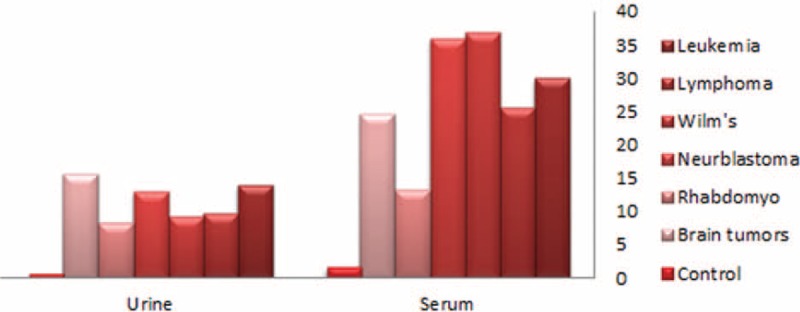
Cadmium status in different cancers versus controls.

No significant differences between the patients and control were observed as regard to age and sex. However, significantly higher percentages of children who suffered from malignancies were exposed to tobacco smoke (72.8% versus 36%, *P* = 0.002), lived under low socioeconomic circumstances (97% versus 77.7%, *P* = 0.002), were raised up in rural areas (87.1% versus 62%, *P* = 0.012) or lived in the vicinity of industrial facility (41.1% versus 4%, *P* < 0.001) when compared with controls as displayed in Table 1. Statistically significant higher Cd levels (serum, urine, scalp hairs, and nails) were observed between cases and controls (*P* < 0.001), detailed description of Cd status in different biologic body samples was displayed in Table 2. Statistically significant higher Cd levels were still detected when comparing each cancer type separately with healthy controls; however, no difference in Cd status, in all samples, could be found between different types of pediatric malignancy as figured out in Table 3 and displayed in Figure 2.

**TABLE 1:**
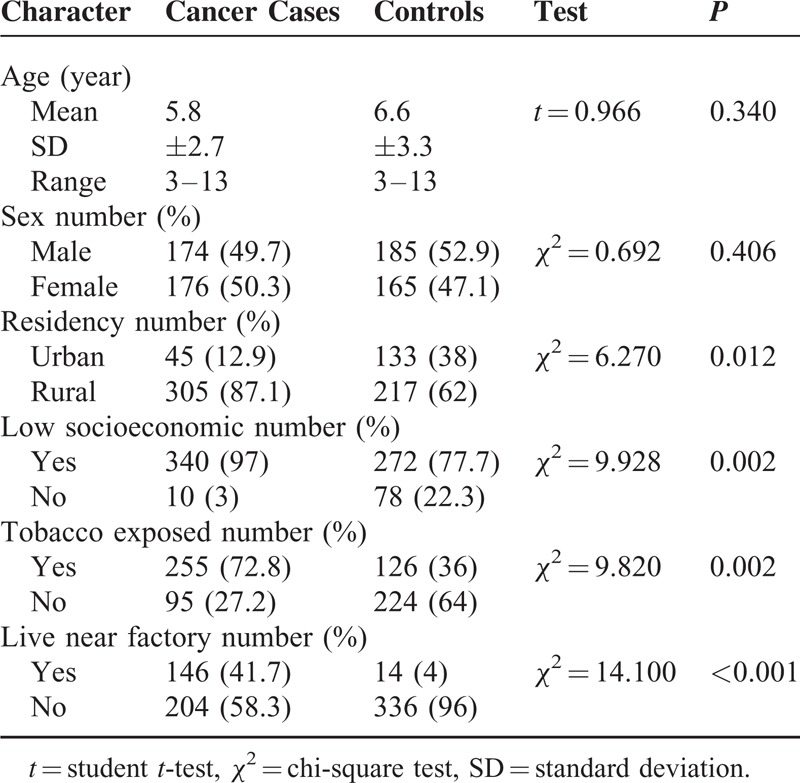
Demographic Characteristics of the Study Groups

**TABLE 2:**
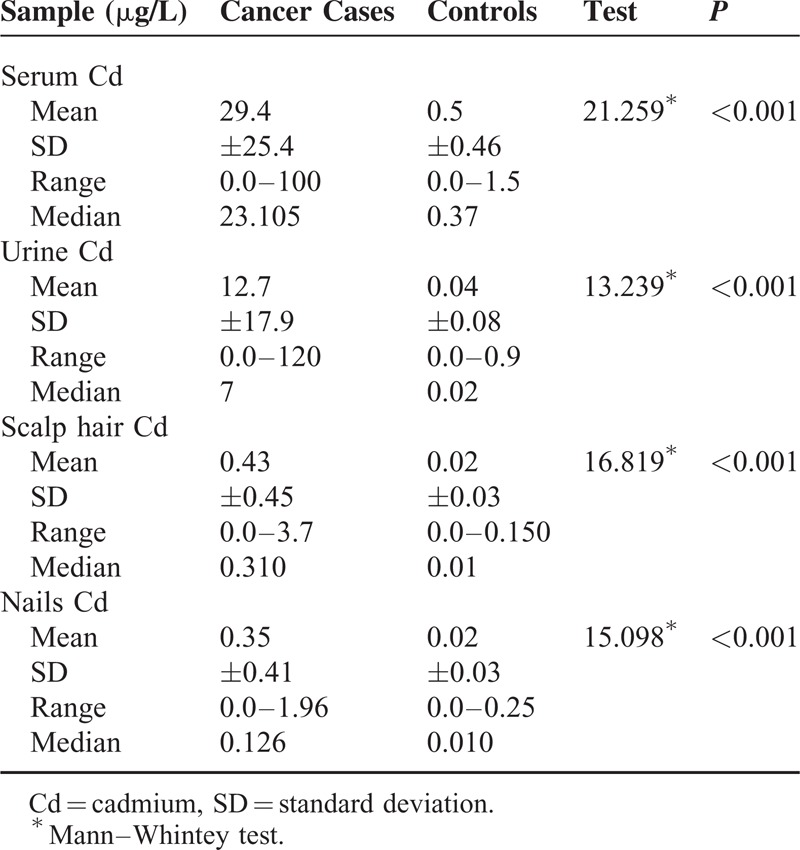
Cadmium Status in Biological Samples of the Study Groups

**TABLE 3:**
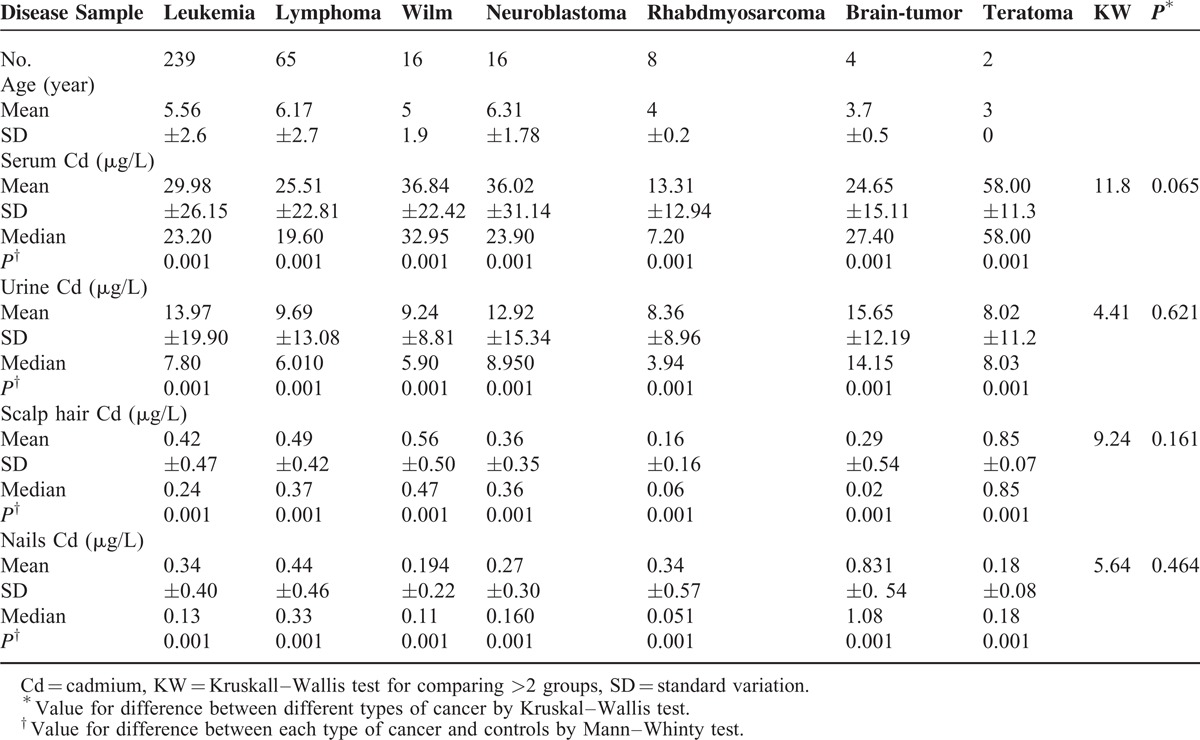
Cadmium Status in Different Types of Malignancy

S-Cd level in pediatric cancer patients who were exposed to tobacco smoke was higher than smoke exposed controls (29.4 ± 27.6 μg/L versus 0.5 ± 0.3 μg/L, *P* < 0.001), also significantly higher S-Cd level was detected in cancer nonexposed cases compared with both exposed and nonexposed healthy controls (7.9 ± 7.4 μg/L versus 0.5 ± 0.3 μg/L, *P* < 0.000 and 0.3 ± 0.1 μg/L, *P* < 0.001), respectively. Similar finding was displayed on interpreting urinary Cd status (exposed cases Cd was 12.7 ± 15.3 μg/L versus 0.04 ± 0.03 μg/L, *P* < 0.001 in exposed controls). Although nonexposed cancer cases U-Cd was 3.4 ± 4.1 μg/L versus 0.04 ± 0.03 and 0.02 ± 0.01 μg/L in exposed and nonexposed comparisons.

## DISCUSSION

In the present cross-sectional case–control study, we tested the hypothesis that pediatric cancer patients are exposed to higher Cd levels than are healthy children with comparable age and sex. Being performed for the first time in pediatric, serum, urine, scalp hair, and nails Cd statuses were assessed as markers of Cd exposure. As Cd accumulates in the human body, particularly at the kidneys,^24^ Cd concentration in urine (U-Cd) is a useful indicator of long-term exposure.^25^ Several studies^22,26,27^evaluated 24 hour urinary Cd excretion or spot urine Cd and depend “solely” upon it as biomarker of life-time exposure. However, Jarup et al^28^ reported that blood Cd may be a better estimate than urinary Cd concentration when tubular damage is present.

In the pediatric population studied here, there were significantly higher Cd levels (serum, urine, hair, and nails) in cancer cases, of all types, when compared with controls, a finding that suggests positive association between body Cd status and malignancy. Such association has been documented by several investigators both in human and experimental animal studies.^12–14,29,32^ Several experimental studies have demonstrated that exposure to Cd initiate benign and malignant tumors at different sites in several species of experimental animals.^29–32^

Regarding possible causal association between Cd exposure and human cancer, the IARC in 1993 concluded that, there was sufficient evidence of carcinogenicity in human for Cd and Cd compounds.^3^ Similar conclusion was released by National Toxicology Program.^29^ Occupational studies have reported an association between Cd exposure and all-cause and cancer mortality including breast, lung, prostate, and renal cancers.^12–14^ However, findings from studies in workers “occupational exposure” should not be extrapolated to the general population as workers in industry are exposed to higher levels of Cd mostly through inhalation, and possibly in the presence of other toxins.^22^ Several general population-based studies in adults have linked Cd to an increased risk of cancer and all cancer mortality.^22,33–39^ Recently, a case–control study, among Japanese women to investigate the relation of urinary Cd and the risk of cancer breast, showed significantly elevated odds ratio of breast cancer for women in the highest tertile of creatinine adjusted Cd level.^26^ Increased urinary Cd was associated with increased risk of pancreatic cancer in South Louisiana;^27^ similar results were documented between S-Cd, as marker of Cd exposure, and early pancreatic cancer in Egyptian population from Mansoura, East Delta.^40^ In contrast to previous results including our, other studies do not found such association,^16,17^ of particular interest is the recent prospective 9 years follow-up cohort study by Sawada et al^15^ who investigated the association between Cd intake and subsequent development of cancer in Japan, they concluded that no association between Cd and cancer at least at the exposure level observed in their population. Possible reasons for this discrepancy might be the route or the range of exposure.

As reviewed in the previously mentioned animal and human studies, high Cd exposure was associated with cancer of different types and locations. In concordance, our results showed significantly higher Cd status in different types of pediatric cancer both hematologic and solid tumors, and also at different sites including CNS, renal, muscles, blood, and lymph nodes. This difference was still detected when comparing each type of malignancy with healthy controls, but of notice, no difference in Cd levels could be observed when comparing different types of malignancy with each other. This observation possibly suggested absence of dose-type relationship between intensity of Cd exposure and type of malignancy but raised the question “Do different routes or duration of exposure elicit carcinogenesis in different systems?” Apart from the inhalation route and cancer lung as target organ for carcinogenesis in occupationally exposed workers,^41^ there is no clear evidences about the possible mechanisms that lead the multiorgan carcinogenicity of Cd.^42^

As Cd is associated with cancer of different types and sites, the mechanisms of carcinogenesis are likely to be multifactorial. First, Cd may act by molecular/atomic mimicry with essential elements, particularly zinc finger motif, that are important in enzyme activity, gene regulation, and maintenance of genomic stability.^43^ Second, Cd is weak genotoxic.^3,30^ Reports of workers exposed to Cd documented a positive correlation between Cd concentration in air and blood, and DNA damage.^44^ Oxidative stress may represent the most important mechanism of Cd-related carcinogenesis as reported by Waisberg et al.^45^ He also suggested that, Cd may have roles in different stages of carcinogenicity. Lastly, Cd also inhibits apoptosis that allows for the accumulation of critical mutation, preneoplastic cells, and even early neoplastic cells.^42,45^ However, there is no direct evidence supporting that Cd causes cancers.

The major sources of Cd exposure in general population “nonoccupational” are foods and tobacco smoke.^3,29^ No single case of active smoking was observed in our study; however, significantly higher percentage of children exposed to household tobacco smokers were found among cancer patients as compared with controls (72.8% versus 36%, *P* < 0.002). Cigarette smoking is a significant source of Cd due to ability of Nicotiana species to concentrate it, 1 cigarette contains 1 to 2 μg Cd.^46^ Our data showed that smoke-exposed cancer cases had significantly higher Cd levels than smoke-exposed controls (*P* < 0.001), and we noticed, also, significantly higher S-Cd level in cancer nonsmoke-exposed cases compared with both exposed and nonexposed healthy controls (*P* < 0.001). These findings may suggest another pivotal source of Cd exposure to cancer cases in our population.

Cd-contaminated food and water may represent the main source of Cd in our region. Rice and wheat are staples of the Egyptian diet, Cd accumulates in grains through uptake of naturally occurring Cd and Cd introduced to the environment through industrial emission, pesticide, and chemical fertilizers. Cd has been detected in high levels in rice in many parts of the world.^47^ Significant increased risk of pancreatic cancer with rice and grain consumption has been documented among Cajuns in south Louisiana, USA;^27^ similarly, Shimbo et al^48^ and Watanable et al^49^ have found a correlation between U-Cd and rice consumption among Japanese women. Previous study done at Dakahlia province, which is geographically located close to our governorate and also share with us the drinking and irrigating water supply from the same river Nile branch, documented high level of heavy metals including Cd and organo-chlorine pesticide in their soil and water.^40^

In the current work, it was observed that higher percentage of children who suffered from malignancy were raised up in rural areas (87.1% versus 62%, *P* = 0.012) and lived under low socioeconomic circumstances (97% versus 77.7%, *P* = 0.002) when compared with control. In concordance with our results, higher percentage of pancreatic cancer patients were living in rural areas compared with controls,^27^ positive association between both S-Cd and farming was documented in pancreatic cancer patient from Dakahlia, Egypt.^40^ A study of metals in household dust conducted at Southern Louisiana found higher mean Cd concentration in both indoor and outdoor dust samples from rural areas than urban.^50^ Children living in rural areas have higher risk due to exposure to fertilizers, pesticides, and also different diet manner with more consumption of grains and leafy vegetables. Low-socioeconomic peoples are at increased risk of Cd toxicity as explained by the fact that nutritional deficiencies especially calcium, zinc, and iron increase Cd absorption by intestine and accumulation in tissues.^51–53^ Nutritional deficiencies especially calcium, zinc, and iron among low-socioeconomic children in our population may represent an important cofactor in Cd accumulation in their bodies and its hazards.

Living in close proximity to industrial facilities may be associated with the variety of health hazards, Cd exposure may be one of it. In the pediatric population studied here, significantly more children with malignancy lived in the vicinity of industrial facility than comparisons (41.1% versus 4%, *P* < 0.001). Urinary Cd level and incidence of lung cancer were highly documented in people who lived close to zinc smelters than those resided far away.^22^

In conclusion, from the indirect evidence currently available about our environment, we have shown a significant positive association between pediatric cancer and environmental exposure to Cd particularly through contaminated food and water. However, further studies are needed to assess Cd levels in our environmental chain (air, soil, and water) and also in different foodstuff to recognize and limit exposure to such toxin.
